# The Impact of Empowering Leadership on Preschool Teachers’ Job Well-Being in the Context of COVID-19: A Perspective Based on Job Demands-Resources Model

**DOI:** 10.3389/fpsyg.2022.895664

**Published:** 2022-05-27

**Authors:** Liying Nong, Jian-Hong Ye, Jon-Chao Hong

**Affiliations:** ^1^Dhurakij Pundit University, Bangkok, Thailand; ^2^School of Education and Music, Hezhou University, Hezhou, China; ^3^Faculty of Education, Beijing Normal University, Beijing, China; ^4^Department of Industrial Education, National Taiwan Normal University, Taipei, Taiwan; ^5^Institute for Research Excellence in Learning Sciences, National Taiwan Normal University, Taipei, Taiwan

**Keywords:** preschool teachers, COVID-19, empowering leadership, job well-being, job stress, perceived organizational support

## Abstract

The COVID-19 epidemic in the early 2020s is making a big difference for educators around the world. For the past 2 years, the curriculum and working patterns have been overturned in response to this epidemic, which has brought unprecedented challenges and physical and mental stress to preschool teachers. This situation can have a drastic impact on the acquisition of job well-being for preschool teachers. During this special time, the leader’s management style will also influence the psychological feelings of the organization’s staff. For example, empowering leadership is an important management function that empowers subordinates, emphasizes the meaning of work, promotes participation in decision-making, and expresses confidence. Therefore, in the current COVID-19 pandemic context, it is worthwhile to explore the topic of empowering leadership to ensure preschool teachers’ well-being, by balancing work demands and work resources in a way that facilitates a sense of organizational support and reduces job stress, while relatively fewer studies have been conducted on the relationship between preschool teachers’ job well-being in the context of the COVID-19 pandemic. Based on this, this study proposed a research model from the theoretical perspective of the Job Demands-Resources Model to explore the influence of empowering leadership, sense of organizational support, and job stress on preschool teachers’ job well-being in the context of the COVID-19 pandemic. To achieve the purpose of this study, a convenience sampling method was used to invite 500 preschool teachers in China to complete a questionnaire survey, and after removing invalid samples and data with incomplete answers, reliability and validity analyses and model fit tests were conducted, followed by a structural equation modeling method for path analysis. The results of the study showed that (1) in the kindergarten work context, empowering leadership showed a significant negative effect on job stress, but a significant positive effect on job well-being and a significant positive effect on sense of organizational support. (2) Sense of organizational support had a significant negative effect on job stress but a significant positive effect on preschool teachers’ job well-being. (3) Preschool teachers’ job stress and job well-being showed a significant negative effect. The contribution of this study was to explore the relationship between understanding leadership empowerment and preschool teachers’ job well-being in the context of the COVID-19 pandemic based on the Job Demands-Resources Model, which will facilitate educational organizational contexts to empower preschool teachers to work harder to reduce their job stress as well as enhance their sense of organizational support and promote the acquisition of job well-being.

## Introduction

The worldwide COVID-19 pandemic in the early 2020s has had a dramatic impact on people’s health, the economy, and society as a whole (Karataş et al., 2021). The COVID-19 pandemic has been ongoing for more than 2 years now and has had a dramatic impact not only on the work and life of people (Supriadi et al., 2020), but also on their physical and mental health and work status (Shah et al., 2022). Many countries and regions have implemented large-scale preventive and emergency measures to cope with the complex environment (Hong J.-C et al., 2021). This has brought great changes to preschool educators around the world, requiring a reversal of previous curricula and work models to respond to this urgent period (Hong X. et al., 2021). Due to the impact of the epidemic, kindergartens continue to repeatedly implement online and offline teaching, the anxiety of many preschool teachers has risen dramatically (Eadie et al., 2021; Yıldırım, 2021). As a result, in addition to the challenging working conditions of the education sector, preschool teachers have to cope with the stresses associated with the impact of the epidemic, including the use of information technology for daily activities, provision of home learning for young children, epidemic prevention and control, and parental guidance (Eadie et al., 2021). These conditions can lead to high levels of job stress as well as burnout and have a drastic impact on preschool teachers’ job well-being (Swigonski et al., 2021).

Furthermore, from the perspective of positive psychology, well-being is seen as a psychological process in which people deal with negative and positive emotions in the face of challenges and changes, cope with crises in a complex and changing environment, and strive for growth and development (Lomas and Ivtzan, 2016). However, in the context of the current COVID-19 pandemic, many preschool teachers are not only at risk for new coronavirus infections, but are also required to undertake tasks such as children’s health information statistics, remote management, and daily teaching activities, placing a great deal of stress and burden on their workload (Duran, 2021). This, coupled with the prevalence of low pay and long working hours among preschool teachers, has in turn led to many problems such as burnout, job stress, and turnover, which are not conducive to high levels of well-being attainment (Li and Zhang, 2019). In addition, Waters et al. (in press) suggested from a positive psychology perspective that during the current COVID-19 pandemic, stress and burden should be reduced through factors such as positive coping or support to promote well-being. Therefore, exploring the enhancement of preschool teachers’ well-being at work and safeguarding the performance and educational quality of the preschool workforce in the context of the COVID-19 pandemic are important issues that need to be addressed today (Thorpe et al., 2020).

In addition, during this special period, the leader’s management style will also influence the psychological feelings and organizational behavior of the organization members (Basuki et al., 2020). For example, empowering leadership, as an important management function, this is therefore an important factor in promoting preschool teachers’ well-being (Liu et al., 2021). Past research has indicated that empowering leadership is strongly associated with teacher well-being, affecting, among other things, teachers’ ability to participate in decision-making and problem-solving on their own (Suleman et al., 2021). In addition, preschool teachers, as important members of kindergarten education institutions, especially during the COVID-19 pandemic, would benefit from more empowerment to increase their intrinsic motivation and positive behaviors (Siswanti and Muafi, 2020), while influencing their work through aspects such as emphasizing the meaning of their work, promoting participation in decision-making status (Bharadwaja and Tripathi, 2021), and to mitigate the negative effects of the epidemic’s impact in a more flexible manner, thus having an impact on preschool teachers’ well-being. Therefore, the purpose of this study was to explore how empowering leadership in the context of COVID-19 affects preschool teachers’ well-being at work.

Moreover, in organizational work research, the Job Demands-Resources Model (JD-R) is often used to explain the relationship between job demands, job resources, and employee well-being in the workplace (Bakker and Demerouti, 2007; Radic et al., 2020). According to the JD-R model, low levels of leadership will likely exacerbate employees’ work demands, while high levels of leadership will likely enrich employees’ work resources, thus affecting organizational outcomes (Schaufeli, 2015). Among them, job stress is considered as a job demand, as it is a negative physiological or psychological reaction caused by people facing organizational job demands (Burman and Goswami, 2018). Whereas job stress may be an important variable in how empowering leadership affects the well-being of preschool teachers. In the current COVID-19 pandemic context, there is a high level of power distance among the Chinese preschool teacher population, who are frequently tasked with instructions from higher leaders, epidemic prevention and control, and parental guidance, placing a high level of work stress on them (Hong X. M. et al., 2021). Empowering leaders are more conducive to shortening the power distance with teachers, giving them more autonomy and vitality, and helping to increase their job satisfaction by relieving job stress and burnout and putting them in a better position to do their jobs (Liu et al., 2021). In addition, researchers have found that during the COVID-19 pandemic, more empowered teachers reduced their job stress and burnout (Collie, 2021), thereby increasing their job well-being (Yu et al., 2021). Therefore, job stress may be a significant predictor variable, and empowering leadership may affect preschool teachers’ job well-being through the pathway of job stress.

Organizational support is an important work resource (Hobfoll, 2011), and organizational support refers to the extent to which employees feel that their well-being is valued and cared about in the organizational environment (Eisenberger et al., 1986). Particularly in the field of education, effective leadership has the potential to positively impact the quality of service and performance of teachers by enriching organizational resources to help them better cope with the complexities of current challenges and changes (Sunarsi et al., 2020). In regards to higher energy, emotional, and professional demands of being affected by COVID-19, preschool teachers are vulnerable to the dual stressors of work and epidemic infection. According to the fact that the work demands they endure cannot be reduced, empowering leadership is more conducive to empowering preschool teachers to increase their energy and sense of organizational support and to engage in their work with more enthusiasm to meet current challenges and difficulties (Liu et al., 2021). In addition, when facing with the challenges and changes of the COVID-19 pandemic, perceived organizational support may help improve well-being at work (Shamsi et al., 2021). Therefore, this study suggests that empowering leadership may affect preschool teachers’ job well-being through the motivational pathway of perceived organizational support.

In summary, in the context of the current COVID-19 pandemic, it is worthwhile to explore the issue of empowering leadership to ensure the well-being of preschool teachers by balancing their job demands and job resources, contributing to perceived organizational support and reducing job stress. However, current research has focused more on the relationship between organizational members’ work stress and organizational performance. Therefore, there is a gap in the research on empowering leadership and teachers’ job well-being in the context of the COVID-19 pandemic. Based on this, this study proposes a research model based on the theoretical perspective of the JD-R model to explore the influence of empowering leadership, perceived organizational support, and job stress on preschool teachers’ job well-being in the context of the COVID-19 pandemic era. Based on the above, this study proposes the following research questions.

RQ1. During the COVID-19 pandemic, did empowering leadership affect the job well-being of preschool teachers?
RQ2. During the COVID-19 pandemic, did empowering leadership affect preschool teachers’ job well-being through job stress?
RQ3. During the COVID-19 pandemic, did empowering leaders influence preschool teachers’ job well-being through perceived organizational support?
RQ4. During the COVID-19 pandemic, did perceived organizational support affect preschool teachers’ job well-being through job stress?

## Research Model and Hypothesis

### Research Model

**Figure 1:**
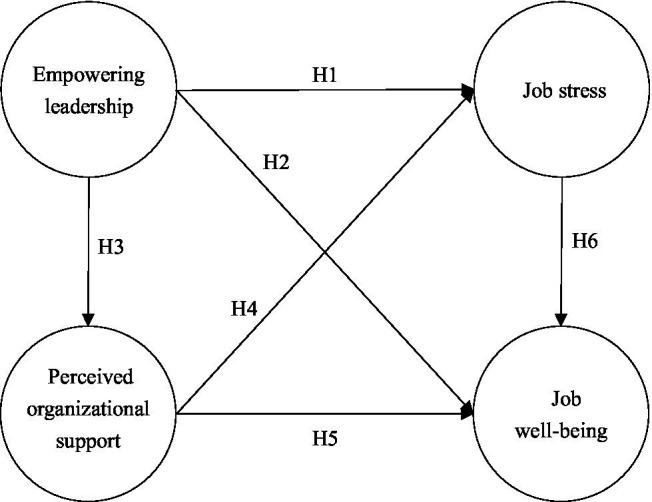
Research model.

The JD-R model assumes that organizational work not only places job demands on employees but also provides them with job resources (Demerouti et al., 2001). According to the JD-R model proposed by Schaufeli (2015), it is believed that leaders should balance employee work demands with resources to maintain healthy, positive, and sustained employee productivity, which helps to reduce work demands as well as enrich work resources to influence organizational outcomes. Therefore, based on the JD-R model, this study explored the relationship between empowering leadership, job stress, perceived organizational support, and job well-being and constructed a research model of empowering leadership affecting preschool teachers’ job well-being in the context of COVID-19, as shown in Figure 1.

### Research Hypothesis

#### The Relationship Between Empowering Leadership and Job Stress

According to Ni (2020), empowering leadership can have a positive impact on employees and the organization, or it can lead to potentially negative impacts. Among other things, research has found that the empowering behavior of leaders in organizations significantly affects employees’ job stress (Tripathi and Bharadwaja, 2020). Furthermore, Syrek et al. (2013) found that leadership behavior is an important factor in balancing employee burnout in organizational work, for example, high levels of empowering leadership may be an important factor in alleviating employees’ autonomy cognitive distractions as well as job tension (Langfred and Moye, 2004), which is beneficial in terms of reducing employees’ job stress (Windeler et al., 2017; Sohn and Kang, 2020). Furthermore, empowering leadership is a leadership style that helps preschool teachers to cope with challenges in complex environments by giving them more power (Liu et al., 2021). In other words, under the influence of COVID-19, it would be detrimental to relieve the work stress and tension of preschool teachers if they are always busy with the arrangement and epidemic control tasks of their supervisors and do not feel the care and empowerment of their leaders (Hong X. M. et al., 2021). Therefore, we proposed the following hypotheses based on the COVID-19 context for empowering leadership and job stress in kindergarten work:

*H1*: Empowering leadership is negatively associated with job stress.

#### The Relationship Between Empowering Leadership and Well-Being at Work

Research has found that higher empowering leadership leads to positive employee outcomes, such as job well-being (Park et al., 2017). Adopting empowering leadership may be a way to promote teachers well-being and inspire positive employee work through enhanced job meaning, thereby contributing to their growth and development (Suleman et al., 2021). In addition, in the field of kindergarten education, empowering leaders can give them more autonomy and flexibility to respond to the challenges and difficulties of COVID-19 rather than just implementing the arrangements and instructions of their superiors (Liu et al., 2021). This will be beneficial in helping preschool teachers to link personal goals as well as organizational goals to promote the acquisition of happiness levels by emphasizing the meaningfulness of work and problem-solving skills (Jiang et al., 2019). When more empowering leadership behaviors are perceived, employees are likely to experience higher levels of well-being (Kim, 2019). Therefore, empowering leadership significantly affects well-being (Premchandran and Priyadarshi, 2018). Therefore, we proposed the following hypothesis on empowering leadership and preschool teachers’ job well-being based on the COVID-19 context:

*H2*: Empowering leadership is positively associated with job well-being.

#### The Relationship Between Empowering Leadership and Perceived Organizational Support

In the JD-R model, leadership can be used to influence organizational outcomes by enriching employees’ job resources (Schaufeli, 2015), and perceived organizational support is used as an important job resource (Hobfoll, 2011). Related research indicates that leaders often influence employees’ organizational behavior and outcomes through organizational resources (Tan et al., 2020). Also, empowering leadership has been suggested to have a positive impact on employees by increasing their intrinsic motivation and well-being through supportive behaviors (Srivastava et al., 2006). Furthermore, studies related to teachers have found that when teachers perceive more empowering leadership behaviors, they will perceive more organizational support, and their job satisfaction will be higher (Bogler and Nir, 2012). In addition, during the COVID-19 pandemic, many preschool teachers were under great physical and mental stress in the face of more demanding workloads (Hong X. M. et al., 2021) and that empowering leadership can enhance their perceived job responsibility through organizational support and can reduce job stress through self-efficacy (Sohn and Kang, 2020). Therefore, there is a positive relationship between empowering leadership and perceptions of organizational support (Naqshbandi et al., 2019). We thus proposed the following hypothesis regarding the relationship between empowering leadership and preschool teachers perceived organizational support in the COVID-19 context:

*H3*: Empowering leadership is positively associated with perceived organizational support.

#### The Relationship Between Perceived Organizational Support and Job Stress

In the JD-R model theory, perceived organizational support is also commonly used to explain negative influences in organizational work situations, which may lead to high levels of job stress when employees have low perceived organizational support (Bakker et al., 2014). In addition, related research has found that a high level of perceived organizational support can reduce conflict in the work environment and improve working conditions, and can, to some extent, alleviate job stress among organizational members (Landells and Albrecht, 2019). In addition to this, past studies have found that perceived organizational support is a significant predictor variable of preschool teachers’ perceived stress, and the more supportive they perceive their supervisors and colleagues to be, the less stressful their jobs are (Masoom, 2021). Also Eksi et al. (2020) stated that organizational support was significantly and negatively related to preschool teachers’ job stress and that perceived organizational support helps to reduce stress and tension in work situations. When faced with the constant dynamic stress of an epidemic, higher perceived organizational support means more help and support for preschool teachers and a greater reduction in job demands and work stress (Yang et al., in press). Xu and Yang (2018) used organizational support theory to explore the relationship between organizational support and teachers’ job stress. Their results indicated that organizational support alleviated teachers’ job stress. Therefore, we proposed the following hypothesis based on the relationship between perceived organizational support and preschool teachers job stress in the context of COVID-19:

*H4*: Perceived organizational support is negatively related to job stress.

#### The Relationship Between Perceived Organizational Support and Job Well-Being

Existing research on employees’ perceived organizational support illustrates that the degree of employee organizational support is related to individual or organizational outcomes (Caesens and Stinglhamber, 2020), such as increased well-being at work (Rasool et al., 2021). According to the JD-R model, perceived organizational support is an important work resource that enhances preschool teachers’ intrinsic positivity and work engagement, and positively influences their well-being (Gang et al., 2018). In addition, in the current COVID-19 pandemic context, enhancing the sense of organizational support for preschool teachers is more conducive to helping them overcome negative effects such as fear and anxiety and job stress (Yang et al., in press). Likewise, preschool teachers who perceive a high level of organizational support will have more energy, focus, and dedication to their work and will make positive contributions to the organization, thus contributing to their well-being (Logan et al., 2021). Therefore, based on the above literature, this study proposes the following hypothesis based on the relationship between perceived organizational support and preschool teachers job well-being in the context of COVID-19:

*H5*: Perceived organizational support is positively correlated with job well-being.

#### The Relationship Between Job Stress and Job Well-Being

Many studies have indicated that job stress has a broad impact on well-being (Tandler et al., 2020; Xie et al., 2021); for example, the JD-R model was used to explain the relationship between teachers’ job stress and well-being (Dicke et al., 2018), which mainly affects their subjective well-being, occupational well-being, and psychological health (Jang et al., 2019). In the field of education, preschool teachers’ job stress not only affects their resources, but also their job positivity and well-being; the higher the job stress, the lower the teachers’ level of well-being (Li and Zhang, 2019). Especially during the COVID-19 pandemic, many preschool teachers have been required to provide additional services and are at risk of infection, leading to high levels of job stress and affecting their well-being acquisition (Eadie et al., 2021). Therefore, this study explored the relationship between preschool teachers’ job stress and job well-being based on the COVID-19 context and proposed the following hypothesis:

*H6*: Job stress is negatively related to job well-being.

## Materials and Methods

### Research Procedure

This study used a convenience sampling method to invite preschool teachers in mainland China to complete a survey. Because of the impact of the epidemic, we used the Wènjuànxīng (WJX) platform, which is one of the most frequently used online survey platforms in China (similar to Google Form). The first page of the questionnaire begins with a description of the purpose of the study, data use, researcher privacy, and informed consent instructions. Those who agreed to complete the questionnaire were considered to be participating in this study. Therefore, the questionnaire for this study was completed with the informed consent of the preschool teachers. In this study, the convenience sampling method was used to invite preschool teachers in mainland China to complete the questionnaire survey. The questionnaires were collected between January and February 2022, and the link to the questionnaires was closed when 500 questionnaires had been received.

### Participants

After deleting the invalid questionnaires with incomplete answers and short response times, the total number of valid data was 453, and the recovery rate was 90.6%. Of these, 441 (97.4%) were female and 12 (2.6%) were male participants; 347 (76.6%) were between the ages of 18 and 30, 76 (16.7%) were between the ages of 31 and 40, and 30 (6.7%) were over 40; 252 (55.6%) were frequent overtime workers and 201 (44.4%) were infrequent overtime workers; and 148 (32.7%) had worked for less than 1 year, 208 (46%) for 2–5 years, 65 (14.3%) for 6–10 years, 18 (4%) for 11–15 years, and 14 (3%) for more than 15 years. There were 283 (62.5%) teaching in public kindergartens and 170 (37.5%) in private kindergartens.

### Measurements

To account for the differences in research contexts, we translated and adapted previous research instruments that had reliability and validity and had undergone three rounds of expert content validity review by three experts in the field of education. Next, 10 preschool teachers were asked to fill in the responses to confirm the face validity of the questionnaire. In addition, to improve the reliability of the responses on the Likert scale, a scale design using a 5-point Likert scale was used, where 1 represents *strongly disagree*, 2 represents *disagree*, 3 represents *neutral*, 4 represents *agree*, and 5 represents *strongly agree*.

#### Empowering Leadership

Empowering leadership is defined as the process of sharing power with employees through a series of specific leadership behaviors that empower employees, promote participation in decision-making, enhance meaningful work, and express confidence (Ahearne et al., 2005). Based on this definition, this study adapted the 12-item Empowering Leadership Scale developed by Ahearne et al. (2005) to assess preschool teachers’ perceptions of empowering leadership during the COVID-19 pandemic. Examples include as: “The director often helps me understand how to link my personal goals to organizational goals” and “The director allows me to make important decisions quickly to meet the needs of children and parents.”

#### Job Stress

Job stress is the physiological or psychological reaction that occurs when people are unable to meet the demands of their jobs (Burman and Goswami, 2018). Based on this definition, we adapted the Teacher Work Stress Scale of Fimian (1984) with eight items to assess the work stress of preschool teachers during the COVID-19 pandemic. Example items include as: “I feel stressed because of the lack of progress at work” and “I feel stressed because I am not in control of the things I do at work related to kindergarten.”

#### Perceived Organizational Support

Organizational support is used to understand the extent to which people perceive that the organization values them and cares about their well-being in the organizational setting (Eisenberger et al., 1986). Based on this definition, we modified the seven-item organizational support scale developed by Eisenberger et al. (1986) to assess preschool teachers’ perceived organizational support during the COVID-19 epidemic. Example items including “My workplace does take care of my welfare” and “My workplace would help me if I had special needs” were used to measure perceptions of organizational support among preschool teachers during the COVID-19 pandemic.

#### Job Well-Being

Well-being at work is defined as people’s positive emotional experiences at work (Orsila et al., 2011). Based on this definition, this study’s questionnaire was adapted from the short version of the Chinese well-being scale by Lu and Lin (2003), which consists of 10 items, to assess the perceptions of preschool teachers about their well-being at work during the COVID-19 epidemic. Example items include as: “I am happy in my work” and “I have a sense of accomplishment in my work performance.”

### Data Analysis

Structural equation modeling (SEM) is widely used to explore the relationship between validated latent variables (Astrachan et al., 2014). Therefore, in this study, SPSS and AMOS software were used to process the data, while the study model was validated by SEM. Item analysis and reliability analysis were first conducted using SPSS, followed by validation factor analysis using AMOS. SEM was used for model fitness analysis and path analysis. Finally, a non-parametric percentile Bootstrap method was used to test for mediation effects (MacKinnon et al., 2004).

## Results and Discussion

### Item Analysis

In this study, first-order factor analysis was used to analyze items for each construct’s items to ensure the internal validity of each construct. First, items with factor loadings (FL) below 0.5 were removed (Hair et al., 2010), followed by a first-order CFA to test the internal validity of each item until the threshold suggested by scholars was reached (Hair et al., 2019). In addition, as can be seen from Table 1, the degree of the proposed summation of the components in this study also meets the criteria of *χ*^2^ values less than 5, *df* less than 5, GFI greater than 0.80, and RMSEA less than 0.1, as proposed by Hair et al. (2019). As a result, the number of items related to empowered leadership decreased from 12 to 8; job stress decreased from 8 to 6; perceived organizational support decreased from 7 to 5; and job well-being decreased from 10 to 8, as shown in Figure 1.

**Table 1:** First-order CFA.

Construct	*χ* ^2^	*df*	*χ*^2^/*df*	RMSEA	GFI	AGFI	FL
Threshold	–	–	<5	<0.10	>0.80	>0.80	>0.05
Empowering leadership	94.5	20	4.726	0.91	0.95	0.91	0.67–0.79
Job stress	25.7	9	2.853	0.64	0.98	0.95	0.66–0.89
Perceived organizational Support	21.4	5	4.282	0.85	0.98	0.94	0.77–0.087
Job well-being	63.5	14	4.536	0.88	0.96	0.91	0.82–0.90

### Reliability and Validity Analysis

To determine the consistency of the study variables, we first used Cronbach’s alpha to conduct a reliability analysis of the questionnaire. Whereas Hair et al. (2019) suggested that a Cronbach’s *α* value of 0.7 and above would be an acceptable threshold, the Cronbach’s *α* value of 0.88 to 0.94 in this study indicated good reliability. Secondly, we conducted a composite reliability (CR) test on the items to determine internal consistency, and from Table 2, it can be seen that the CR values in this study ranged from 0.91 to 0.95, which were above 0.7, which is in line with the criteria suggested by scholars (Fornell and Larcker, 1981). In addition, we used the Average Variance Extracted (AVE) test to determine the convergent validity, and as shown in Table 2, the AVE values in this study ranged from 0.56 to 0.73, all of which were greater than 0.5, and the AVE values for each construct met the criteria suggested by scholars (Hair et al., 2011).

**Table 2:** Reliability and validity analysis.

Construct	M	SD	*α*	FL	CR	AVE	*t*
Empowering leadership	3.35	0.718	0.92	0. 75	0.91	0.56	13.71–16.30
Job stress	3.36	0.88	0.92	0. 82	0.92	0.67	14.55–15.93
Perceived organizational support	3.31	0.754	0.89	0. 82	0.91	0.68	19.79–23.90
Job well-being	3.39	0.749	0.94	0. 85	0.95	0.73	21.21–24.42

### Model Fit Analysis

To confirm the overall fitness of the study model, it was tested using the AMOS 25.0 statistical software. Structural equation models should take into account multiple fitted statistical indicators (Thompson, 2000), and scholars have suggested that the value of *χ*^2^/*df* must be less than 5 (Hair et al., 2010). The RMSEA should be less than 0.1; the values of GFI, AGFI, NFI, NNFI, CFI, IFI, and RFI should be greater than 0.800 (Abedi et al., 2015), while the values of PNFI and PGFI should be greater than 0.500 (Hair et al., 2010). In the present study, *χ*^2^ = 649, *df* = 293, *χ*^2^/*df* = 2.215, RMSEA = 0.05, GFI = 0.90, AGFI = 0.88, NFI = 0.93, NNFI = 0.96, CFI = 0.96, IFI = 0.96, RFI = 0.93, PNFI = 0.84, and PGFI = 0.75, all of which meet the criteria recommended by scholars and so have good model fit.

### Path Analysis

**Figure 2:**
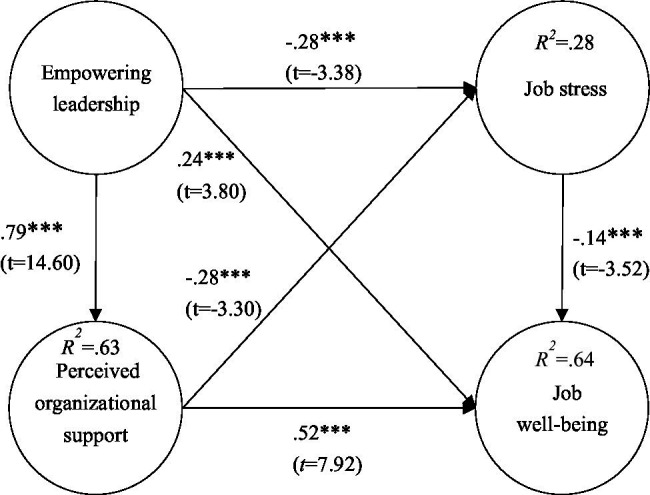
Validation of the research model. ^***^p < 0.001.

Model validation results show that empowered leadership had a negative effect on job stress (*β* = −0.28***; *t* = −3.38); Empowered leadership had a positive effect on job well-being (*β* = 0.24***; *t* = 3.80) and perceived organizational support (*β* = 0.79***; *t* = 14.60); Perceived organizational support had a negative effect on job stress (*β* = −0.28***; *t* = −3.30); Job stress had a negative effect on job well-being (*β* = −0.14***; *t* = −3.52); and Perceived organizational support had a positive effect on job well-being (*β* = 0.52***; *t* = 7.92), as shown in Figure 2.

According to Hair et al. (2010), when the explanatory power values are in the range between 0.25, 0.50, and 0.75, they represent weak, medium, and strong degrees of explanatory power, respectively. In this study, the explanatory power of job stress was 28%, the explanatory power of organizational support was 63%, and the explanatory power of job well-being was 64%, indicating that this study had explanatory power of weak, moderate, and strong degrees, as shown in Figure 2.

### Indirect Effect Analysis

From the bootstrapping results, it is clear that in terms of indirect effects, empowered leadership is indirectly and significantly positively related to job well-being (*β* = 0.24***) with a confidence interval of [0.37,0.60]; Job stress was indirectly and significantly negatively related to job well-being (*β* = −0.14***) with a confidence interval of [−0.22,-0.06]; and Perceived organizational support was indirectly and significantly positively related to job well-being (*β* = 0.52***) with a confidence interval of [0.01,0.08], 95% of the confidence intervals did not contain 0, as shown in Table 3.

**Table 3:** Indirect effect analysis.

Construct	Empowering leadership		Job stress		Perceived organizational support	
	*β*	95% CI	*β*	95% CI	*β*	95% CI
Job well-being	0.24^***^	[0.37, 0.60]	−0.14^***^	[−0.22, −0.06]	0.52^***^	[0.01, 0.08]

***p < 0.001.

### Discussion

The COVID-19 pandemic has created great challenges in the field of educational organizations, forcing many of them to change in different ways (Basuki et al., 2020). In contrast, the JD-R model emphasizes that leadership should mitigate the work demands of employees when faced with challenges and provide organizational resources to influence organizational outcomes (Schaufeli, 2015). Therefore, this study explored the influence of empowering leadership on preschool teachers’ job well-being based on the JD-R model in the context of the COVID-19 pandemic.

#### Empowering Leadership Negatively Predicted Job Stress

As COVID-19 continues to explode, leadership, a key factor in responding to the crisis, is needed to reduce work tensions by increasing individual job autonomy and team cohesion to impact organizational performance (Bartsch et al., 2021). According to the JD-R model, leadership reduces job burnout and positively influences job well-being by reducing teachers’ job demands (Adil and Kamal, 2020). Liu et al. (2021) indicated that empowering leadership as an important leadership style and giving more power to preschool teachers and could alleviate job stress and tension to some extent and positively affect their job satisfaction through organizational commitment. Also, the study by Tripathi and Bharadwaja (2020) found that empowered leadership was negatively associated with job stress and affected people’s overall psychological well-being. In addition, the anxiety and stress of preschool teachers have increased dramatically due to the impact of the COVID-19 pandemic, and empowered leaders can reduce the negative impact on preschool teachers and help them better cope with the crisis and stress of the pandemic to promote quality infant and child care (Bauwens et al., 2021). The results of this study indicated that empowered leadership was negatively associated with preschool teachers’ job stress, which is consistent with previous research. That is, when preschool teachers perceive higher empowerment, they will feel a lower level of job stress.

#### Empowering Leadership Positively Predicted Well-Being at Work

According to Srivastava et al. (2006), empowering leaders emphasize enhancing work meaning and autonomy, provide supportive behaviors to motivate employees, and have a significant positive impact on employees’ job positivity (Ghadi and Almanaga'h, 2020). Also, a study by Park et al. (2017) indicated that empowered leadership is a significant predictor of well-being, positively influencing people’s work and lives through motivational pathways. In the field of educational organization, when preschool teachers are given more power, they are psychologically more satisfied to comply with kindergarten order and requirements and experience higher levels of well-being at work (Liu et al., 2022). In addition, Jiang et al. (2019) also pointed out that if kindergarten leaders can empower preschool teachers to be more empowered, they cannot only meet their needs in terms of decision-making participation, autonomy, and professional growth, but also motivate them to work, which can, to some extent, increase their job satisfaction and thus promote the acquisition of job happiness. Therefore, the results of this study, based on the COVID-19 pandemic context, indicated that empowered leadership was positively associated with preschool teachers’ job well-being, which is consistent with previous studies. That is, when preschool teachers perceive higher levels of empowered leadership, they will perceive higher levels of job well-being.

#### Empowered Leadership Positively Predicted Perceived Organizational Support

According to the JD-R model, leadership can influence organizational outcomes by enriching organizational resources (Tan et al., 2020). Hellman et al. (2006) identified organizational support as an important organizational resource that predicts better job performance, less job stress, and organizational outcomes in the face of crisis. Kim et al. (2018) also indicated that high levels of empowered leadership were found to have a significant impact on employees’ perceived organizational support in previous studies. Especially in the field of education, Oubibi et al. (2022) noted that many teachers are experiencing high levels of job stress and burnout as a result of the global COVID-19 pandemic, and to address this challenge, educational leaders should provide more organizational support to teachers to increase their job positivity and responsibility, thereby improving positive teacher behavior and job satisfaction. Also, the study by Jiang et al. (2019) supports the idea that as kindergarten leaders, they should empower preschool teachers to provide a supportive work environment and conditions to enhance their perceptions of the meaning and autonomy of their work and be in a better position to deal with a variety of challenges and difficulties. Therefore, the results of this study showed that empowered leadership was positively related to preschool teachers perceived organizational support, which is consistent with past research. That is, when preschool teachers perceive higher empowered leadership, they will perceive a higher level of organizational support.

#### Perceived Organizational Support Negatively Predicted Job Stress

According to the JD-R model, organizational support is considered an important variable in work situations and is often used to ameliorate negative influences in organizational settings, such as stress as well as burnout (Bakker et al., 2014). A study by Hege et al. (2019) found that employees who perceived a high level of organizational support tended to have lower levels of anxiety and burnout because the more support they perceived, the more they could increase their job positivity, which then influenced their behavior and outcomes. Moreover, Trinidad (in press) pointed out that during the COVID-19 pandemic, teachers endured more load and burnout and that increasing the perceived organizational support for teachers at the school level would be beneficial in terms of reducing burnout and thus increasing job satisfaction. In addition, Ji and Yue (2020) also indicated that preschool teachers often face pressure from parents and young children, and that more organizational support would help to balance their work demands and resources, thus further alleviating the negative effects of work stress and burnout. Therefore, the results of this study indicated that the perceived organizational support negatively predicted preschool teachers’ job stress. That is, when preschool teachers perceive higher levels of organizational support, they will perceive lower levels of job stress.

#### Perceived Organizational Support Positively Predicted Job Well-Being

Duran (2021) noted that as a result of the COVID-19 pandemic, many preschool teachers have had to cope with the additional stress of the pandemic on top of their daily busy workloads, seriously jeopardizing their mental health and well-being. In addition, according to Oubibi et al. (2022), when faced with the crisis and challenges of the epidemic, organizational support will help teachers to meet the demands of their work, increase their work engagement, and contribute to their career satisfaction. In other words, more organizational support would be beneficial to enhance preschool teachers’ well-being at work. For example, Cassidy et al. (2017) stated that providing a supportive work environment and conditions for preschool teachers and giving them more care would be conducive to relieving work stress and engaging in work with more positive emotions and more energy, thus promoting high levels of work well-being. Moreover, the study by Wattoo et al. (2018) confirmed that the feeling of organizational support was significantly and positively related to job well-being. Therefore, the results of this study are consistent with previous studies. That is, when preschool teachers perceive higher levels of organizational support, they will perceive a higher level of well-being at work.

#### Work Stress Negatively Predicted Job Well-Being

Clabaugh et al. (2021) suggested that people were particularly at risk of depression and anxiety during the COVID-19 pandemic. Similarly, Hong X. M. et al. (2021) suggested that preschool teachers are at particularly high risk of poor mental health outcomes when affected by an epidemic. Li and Zhang (2019) also suggested that preschool teachers often face a variety of challenges, which can result in high levels of job stress, and negatively affect job well-being. According to Eadie et al. (2021), during the continued global outbreak of COVID-19, many preschool teachers have been exposed to high stress workloads in addition to the risk of infection, among other things, which seriously affects their job well-being (Tebben et al., 2021). The results of the present study indicated a negative association between preschool teachers’ job stress and job well-being in the context of the COVID-19 pandemic, which echoes previous studies. That is, when preschool teachers perceive lower levels of job stress, they will perceive a higher level of job well-being.

## Conclusion and Recommendations

### Conclusion

Leadership, as an important management function in the organizational field, plays an important role in promoting organizational behavior and performance. Therefore, exploring the impact of leadership in the organizational sphere will help enrich organizational resources to achieve goals and performance. However, with the global COVID-19 pandemic, the impact of leadership on the field of educational organizations is becoming an increasingly widely discussed topic. In addition, many preschool teachers have been subject to many additional workloads as a result of COVID-19, and empowering leadership will facilitate a stronger connection between the organization and teachers to better cope with the stresses and challenges. In addition, the JD-R model, which explains the effects of job characteristics on organizational outcomes, has been widely used in the organizational field. However, it is less frequently mentioned in the field of kindergarten education organization.

Therefore, this study takes the perspective of organizational psychology that leadership is a key factor in coping with crisis and enhancing well-being. The results of the study found that in the context of the COVID-19 pandemic, higher levels of empowering leadership were associated with lower levels of job stress and higher levels of job well-being and perceived organizational support among preschool teachers. Therefore, under the current severe COVID-19 pandemic challenges, empowering leaders play a critical role in kindergarten education organizations by empowering kindergarten teachers with more power, meaningful work and confidence to alleviate the dual stress of their daily and epidemic prevention work. In addition, when preschool teachers perceive higher levels of organizational support, they will have lower levels of job stress and higher levels of job well-being. Therefore, empowering leadership as an effective leadership is more conducive to helping preschool teachers link their personal goals to organizational goals, motivate and guide them to better achieve their mission through organizational support, and thus sustain a high level of well-being. In addition, when preschool teachers’ job stress was lower, their level of job well-being was higher. This suggests that kindergarten administrators should promote a positive and stable state of well-being among preschool teachers through motivational tools such as organizational support.

The results of this study will help expand the application of empowered leadership in the field of educational organizations under the framework structure of the JD-R model. This study found that in the context of the COVID-19 pandemic, empowering leaders can reduce preschool teachers’ job stress through stress pathways and also enrich preschool teachers perceived organizational support through motivational pathways, thereby affecting preschool teachers’ job well-being.

### Recommendations

According to the above findings, in the context of the COVID-19 pandemic, empowered leadership is critical to job well-being, affects job stress, and enhances perceived organizational support. Past research has found that empowered leaders, by giving more authority, can help employees overcome challenges in complex environments, meet job demands, and reduce the negative effects of job stress. Therefore, it is recommended that leaders in kindergarten education organizations should focus on empowering preschool teachers and providing more autonomy to help them deal with various pressures and challenges, thereby enhancing their sense of well-being at work.

According to the theory of the JD-R model, the organizational results of employees in organizational work interact with their job demands, organizational resources, and other factors. Empowering leaders who promote participation in decision-making, emphasize the meaning of work, and express confidence in order to increase employees’ perceptions of organizational support and motivate them to work positively will contribute to a greater sense of well-being at work. The findings of this study confirm that the higher the perceived empowered leader, the higher his or her perceived organizational support. Perceived organizational support, an important organizational resource, is even more beneficial in the current COVID-19 pandemic context to strengthen leaders’ connections with teachers, balance job demands with resources, and subsequently feel a higher level of well-being. Therefore, it is suggested that in dealing with the complex changes and challenges of COVID-19, kindergarten leaders should emphasize the positive role of empowering leadership, give more organizational resources and support to preschool teachers, and provide more support in the form of goal leadership, professional development, working conditions, and emotional management, thus promoting work well-being.

### Limitations and Future Study

According to the JD-R model, leadership can influence organizational outcomes through different pathways (Schaufeli, 2015). Moreover, according to Fahlevi (2021), when good working environment and conditions are provided, it not only helps employees to generate motivation and enthusiasm for their work, but also has a positive impact on their performance and performance. Furthermore, the study by Sembiring et al. (2021) indicated that leadership may affect employee performance and job satisfaction through different organizational cultures. Especially in the face of crises and challenges, organizational culture is beneficial in helping leaders of educational institutions to motivate and guide teachers, thus positively influencing job performance and organizational outcomes (Hasibuan, 2022). Therefore, the influence of different organizational cultures on empowering leadership and job well-being may be explored in a follow-up study.

In addition, the post-epidemic era refers to the era of normalization of the epidemic in which the new coronavirus and people have been fighting and coexisting for a long time. In the post-epidemic era, although the tension caused by the epidemic in most countries or regions can be alleviated to a certain extent, there will be small-scale outbreaks, localized epidemic prevention and control, and disruption of people’s economy and life. Meanwhile, the post-epidemic era not only brings a great burden to people’s work and life, but also may endanger their psychological health and sense of well-being. Therefore, how to maintain a sense of well-being in the post-epidemic era is an issue worth discussing. Thus, the state of preschool teachers’ well-being in the post-epidemic era may be explored in subsequent studies to explore the motivational effects of promoting preschool teachers’ well-being through different perspectives.

In addition, researchers have found qualitative studies to be beneficial in explaining some complex structural phenomena and more beneficial in explaining the interactions between variables over time (Lindgren et al., 2020). However, this study was conducted mainly in a cross-sectional manner, and therefore is not able to provide deeper insights into the impact of empowering leadership and its influencing factors on preschool teachers’ job well-being. Qualitative interviews may be used in future studies to understand preschool teachers’ perceptions of empowering leadership and job well-being in order to further extend the findings.

## Data Availability Statement

The raw data supporting the conclusions of this article will be made available by the authors, without undue reservation.

## Ethics Statement

Ethical review and approval were not required for the study on human participants in accordance with the local legislation and institutional requirements. Written informed consent for participation was not required for this study in accordance with the national legislation and the institutional requirements.

## Author Contributions

LN and J-HY: concept and design, drafting of the manuscript, acquisition of data, and statistical analysis. LN and J-CH: critical revision of the manuscript. All authors contributed to the article and approved the submitted version.

## Conflict of Interest

The authors declare that the research was conducted in the absence of any commercial or financial relationships that could be construed as a potential conflict of interest.

## Publisher’s Note

All claims expressed in this article are solely those of the authors and do not necessarily represent those of their affiliated organizations, or those of the publisher, the editors and the reviewers. Any product that may be evaluated in this article, or claim that may be made by its manufacturer, is not guaranteed or endorsed by the publisher.
